# Validation of a 3D perfused cell culture platform as a tool for humanised preclinical drug testing in breast cancer using established cell lines and patient-derived tissues

**DOI:** 10.1371/journal.pone.0283044

**Published:** 2023-03-16

**Authors:** Peng Liu, Sophie Roberts, James T Shoemaker, Jelena Vukasinovic, Darren C Tomlinson, Valerie Speirs

**Affiliations:** 1 Institute of Medical Sciences, University of Aberdeen, Aberdeen, United Kingdom; 2 Leeds Institute of Medical Research at St James’s, University of Leeds, Leeds, United Kingdom; 3 Lena Biosciences, Atlanta, GA, United States of America; 4 School of Molecular and Cellular Biology, University of Leeds, Leeds, United Kingdom; Teikyo University, School of Medicine, JAPAN

## Abstract

3D cell culture models of cancer are currently being developed to recapitulate *in vivo* physiological conditions and to assess therapeutic responses. However, most models failed to incorporate the biochemical and biophysical stimuli from fluid flow. In this study, a three-dimensional scaffold, SeedEZ was applied within the PerfusionPal perfused culture system to investigate how perfusion, and blood-like oxygen delivery influenced breast cancer cell growth and their responses to a commonly used breast cancer drug tamoxifen. Our results showed that breast cancer cells could be maintained over 3 weeks in PerfusionPal with increased cell viability compared to static 3D culture in fully humanised conditions. This platform also supported examining the effect of tamoxifen on breast cancer cell lines and in primary patient-derived breast cancer samples. Future work is warranted to further the adaption for fully humanised assessment of drug effectiveness in a patient personalized approach with the aim to reduce the burden of animal use in cancer research and increase the degree of human pre-clinical data translation to clinic.

## Introduction

For decades pre-clinical cancer research has relied on two principal research tools to assess response to potential anti-cancer drugs: 2D cell culture using established cell lines, and animal models including patient derived xenografts [1]. There are examples of success using these types of approaches, notably tamoxifen (TAM), arguably one of the most successful hormone therapies ever developed to treat estrogen receptor (ER)-positive breast cancer (BC), which used both type of pre-clinical models [2]. However, the current success rate in translating pre-clinical findings into the clinic is around 5% [3, 4]—partly attributed to drug response data obtained from 2D cell culture where cells are grown on a plastic substate which is missing tissue-specific mechanical/biochemical signals from the microenvironment which can influence cell polarization, differentiation, proliferation, apoptosis, metabolism, gene/protein expression, etc [5, 6]. Indeed 2 industry reports covering 2006–2015 and 2011–2020 time periods showed that out of 14 major disease areas, oncology had the lowest overall likelihood of approval (LOA) from Phase I and there was no improvement over the last 10 years apart from a rare pocket of success from immune-oncology [7, 8]. There is also the question of the relevance of using animal models to study human disease [9, 10]. Consequently, the need to shift from traditional 2D cell culture systems, at the same time being mindful of the 3Rs (Replacement, Reduction, and Refinement), in research is increasingly being recognised not only by the research community, but also by governments and biotechnology industries [11]. Collectively, the relatively low degree of translation of pre-human data into the clinic, increasing awareness of the United Nations’ Sustainable Development Goals (SDGs) with respect to animal welfare [12] and increased adoption of the 3Rs highlight the need for next-generation human-relevant pre-clinical tumour models that can reduce drug attrition in the clinic while meeting SDGs.

More contemporary models include 3D cell culture models which often contain multiple cell types to recapitulate the cell-cell interactions seen in the tumour microenvironment (TME) and some have been used for phenotypic screening in BC [13–15]. Patient-derived organoid models pioneered by the Clevers laboratory are also becoming routine [16] as these better reflect what is observed in patients. Using patient-derived samples has the added benefit of helping reduce the numbers of animals used in research, addressing the 3Rs directly. However, many of these new approaches still rely on static culture, with cells grown in culture media, often supplemented with growth factors and fetal bovine serum (FBS), an animal derivative. Considering that most tumours bigger than approximately 2 mm are perfused and that the access to nutrients and oxygen via blood supply is required for tumour growth and metastasis [17–19], perfused tumours *in vitro* may be able to better model a tumour the patient has at the time of drug treatment.

Recently a new platform, PerfusionPal, was described [20]. This organ-on-a-chip insert system comprises a scaffold, SeedEZ, composed of randomly arranged inert glass microfibres housed within a 12-well plate, to which oxygen is delivered to cells or tissues growing in 3D, via a breathable haemoglobin analogue. The aim of this study was to use PerfusionPal to deliver a fully humanised method of breast cancer cell culture and to examine the feasibility of this platform in supporting the growth of patient-derived human breast cancer tissue.

## Materials and methods

### Ethics statement

Primary BC cells were obtained as explants from the Breast Cancer Now Tissue Bank (http://www.breastcancertissuebank.org) following patient consent. Clinical information for these samples is detailed in Table 1 (the patient ID number is the pseudonymised laboratory code). Ethical approved was obtained from Cambridge Central Research Ethics Committee (21/EE/0072). Written informed consent was obtained for the use of the patient-derived tissues described in this paper.

**Table 1:** Clinical information of patient samples.

Patient ID	Age	Grade	ER status (score)	HER2 status (score)
1681	44	2	+ (8)	—(1+)
1756	60	3	+ (5)	+ (3+)
2400	37	3	+ (7)	+ (3+)
2724	68	3	+ (8)	+ (3)

### Culture of cell lines and patient-derived samples

MCF-7, BT-474, and MDA-MB-231 BC cell lines were purchased from ATCC and maintained under recommended culture conditions (S1 Table). Cells underwent regular mycoplasma testing (consistently negative) and provenance was verified by short-tandem repeat (STR) profiling (NorthGene, Newcastle, UK; last tested May 2021). For fully humanized culture conditions, we used FibroLife (Lifeline Cell Technology, Frederick, USA), a defined medium optimized for serum-free culture, which supported the growth of BC cell lines as described previously [14]. Patient-derived explants were cultured in Advanced DMEM-F12 (ThermoFisher Scientific, Loughborough, UK) with recommended supplements [16].

### 3D cell culture in SeedEZ and PerfusionPal

We used the PerfusionPal insert system (Lena Biosciences, Atlanta, USA), which has been described previously [20]. In brief, this is an integrated multi-well insert with SeedEZ scaffolds (Lena Biosciences, Atlanta, USA) placed within the wells of PerfusionPal system. SeedEZ scaffolds are clear, hydrophilic disks comprising randomly arranged glass fibres. BC cell lines or primary explant cultures were plated onto SeedEZ disks and cultured either under static or perfused conditions. Perfusion was achieved by a proprietary in-well perfusion method where a high-density ‘Blood Substitute’, a breathable haemoglobin analogue (Lena Biosciences, Atlanta, USA) was delivered by a syringe pump set to infuse and withdraw 10 mL twice over a 24-hour period (flow rate 0.028 mL/min). Half media changes were carried out every other day. Cells/explants were cultured for at least 5 days and measurements made as described below. A diagram of the platform is shown in S1 Fig.

### Assessment of metabolic activity

Cell metabolic activity was measured with the AlamarBlue assay (BioRad, Watford, UK) according to the manufacturer’s instructions. Samples were incubated with 10% AlamarBlue at 37°C, 5% CO_2_ until a colour change was visible. Fluorescence intensity was obtained at 540 nm excitation and 590 nm emission (PHERASTAR FS plate reader; BMG Labtech).

### Cell viability

To indicate viable and dead cells simultaneously in the SeedEZ scaffold, the LIVE/DEAD® Viability/Cytotoxicity Kit for mammalian cells (ThermoFisher Scientific, Loughborough, UK) was used. As per the manufacturer’s instructions, 1 μM calcein AM and 2 μM ethidium homodimer-1 (EthD-1) were used to stain cells in scaffolds for 30 mins. Metabolic conversion of calcein AM by intracellular esterase activity to calcein (ex/ex around 495/515 nm) indicates viable cells. As an indicator of dead cells, EthD-1 enters cells with damaged membranes and binds to nucleic acids leading an enhancement of fluorescence (ex/ex around 495/635 nm). After staining, scaffolds were immediately transferred to a slide and imaged (Zeiss Axio Observer Z1 inverted microscope). As MCF-7 cells had been pre-labelled with green fluorescent protein (GFP), cell viability was also measured with direct fluorescence (485 nm excitation, 520 nm emission) within a black plate (Ibidi, Gräfelfing, Germany).

### Response to TAM

TAM (Fisher scientific, Loughborough, UK) was dissolved in ethanol. Cells were seeded on SeedEZ with or without PerfusionPal for 1 day before the start of exposure. Prior to exposure, baseline viability was determined by fluorescence intensity. Drugs were then added with half-media change to reach a final concentration of 0–100 nM. The final vehicle concentration in the medium during exposure was 0.1% v/v. Cultures were then incubated at 37°C, 5% CO_2_ with humidity for up to 21 days in either static or perfused conditions as described above with half-media change with treatment (vehicle or drug) every other day.

### Statistical analysis

All experiments were performed at least in triplicate. 2-way ANOVA with Tukey’s multi-comparison post-test or Students t-test was performed (Prism 6; GraphPad Software, Inc., La Jolla, USA) and graphs created. P < 0.05 was considered statistically significant.

## Results

### Comparison of cell behaviour and metabolic activity under static and perfused conditions

**Fig 1:**
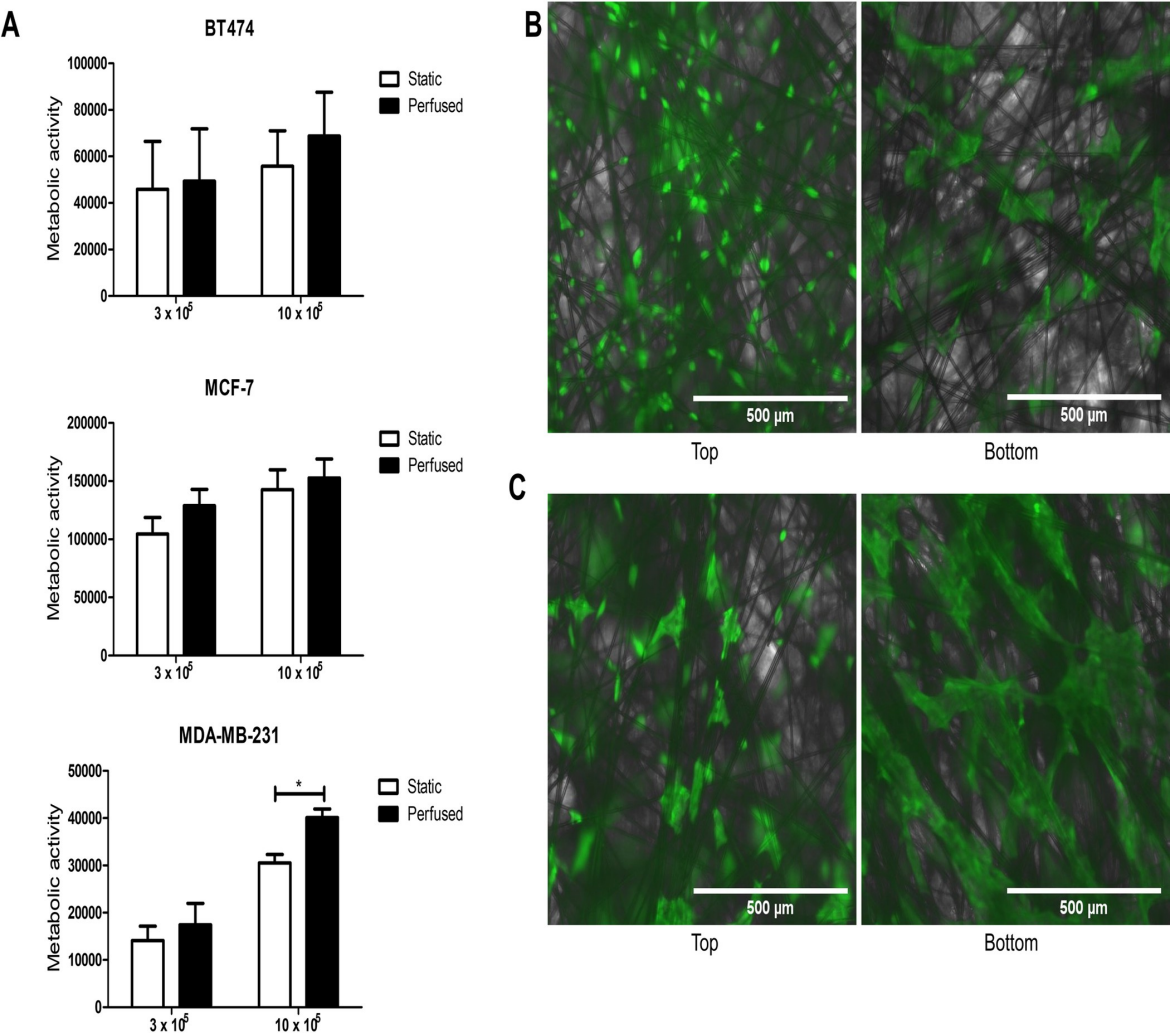
Metabolic activity of breast cancer cell lines under static or perfused conditions. Cell lines were cultured for 5 days either in static (open bars) or perfused (solid bars) conditions and metabolic activity determined using the AlamarBlue assay (A). Representative images from BT-474 cells grown in SeedEZ with different densities for 5 days and stained with Calcein AM in either (B) static or (C) perfused conditions. Images were taken on an EVOS fluorescent microscope (x4 magnification). Scale bar = 500 μm. Data represents the mean ± SD (N = 3). Statistical significance between static and perfused conditions, *p < 0.01.

We used a panel of 3 cell lines representative of the different molecular classes of BC [21] to investigate if there were differences in cell behaviour under static or perfused culture for up to 5 days. At higher seeding densities, there was generally greater metabolic activity in cells cultured under perfused compared to static conditions (Fig 1A), especially with MDA-MB-231 (P < 0.01). Using BT-474 as an example, perfusion appeared, at least by visual inspection, to result in more even cell growth across the SeedEZ compared to those grown in static conditions (Fig 1B and 1C).

### Perfusion improves cell metabolic activity in fully humanised cell culture conditions

**Fig 2:**
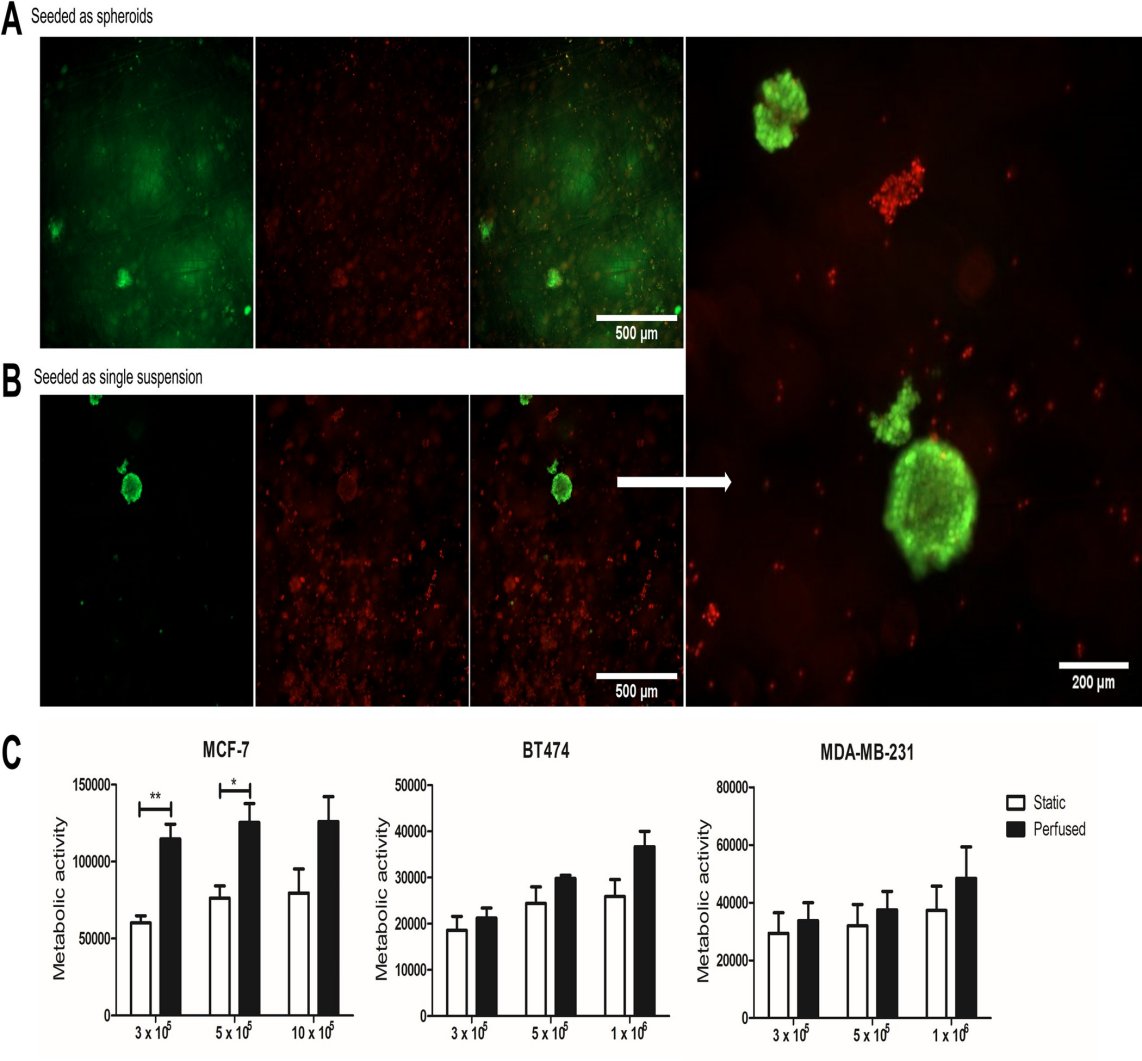
Metabolic activity of breast cancer cell lines under static or perfused conditions when cultured in fully humanised media. MCF-7 cells were seeded onto SeedEZ as (A) pre-formed spheroids or (B) single cell suspensions in epiFL. After 5 days, cells were stained with calcein-AM (green) and EthD-1 (red) and imaged on a Zeiss Axio Observer Z1 inverted microscope. Scale bar as indicated. (C) Quantification of the metabolic activity in different cell lines using AlamarBlue at day 5 cultured either in static or perfused conditions using modified FibroLife. Results represents the mean ± SD (N = 3). Statistical significance between static and perfused conditions. *p < 0.05, **p < 0.01.

Previously we demonstrated that BC epithelial cells grew spontaneously as spheroids in a modification of a fully humanised culture medium, FibroLife, renamed epiFL [14]. Hence, we wished to explore how BC cell lines cultured in this media performed in perfused versus static conditions. When cells were seeded onto SeedEZ as pre-formed spheroids of 300–600 μm in diameter (Fig 2A) they were unable to penetrate through the scaffold interior. Next we added cells in a single cell suspension of epiFL (Fig 2B). Consistent with our previous study [14], cells seeded as a single cell suspension on SeedEZ formed spheroids spontaneously, as judged by green calcein-AM fluorescence. Not all cells formed spheroids; dead/dying cells were seen, indicated by red EthD-1 fluorescence. Having established that pre-formed spheroids were unsuitable, we seeded single cell suspensions of MCF-7, BT-474 and MDA-MB-231 cultured in epiFL [14] into SeedEZ. Metabolic activity was measured by AlamarBlue at day 5. Higher metabolic activity was seen under perfusion, however this was only significant with MCF-7 (nearly 2-fold-fold compared to static culture) (Fig 2C).

### Perfusion improves proliferation over time

**Fig 3:**
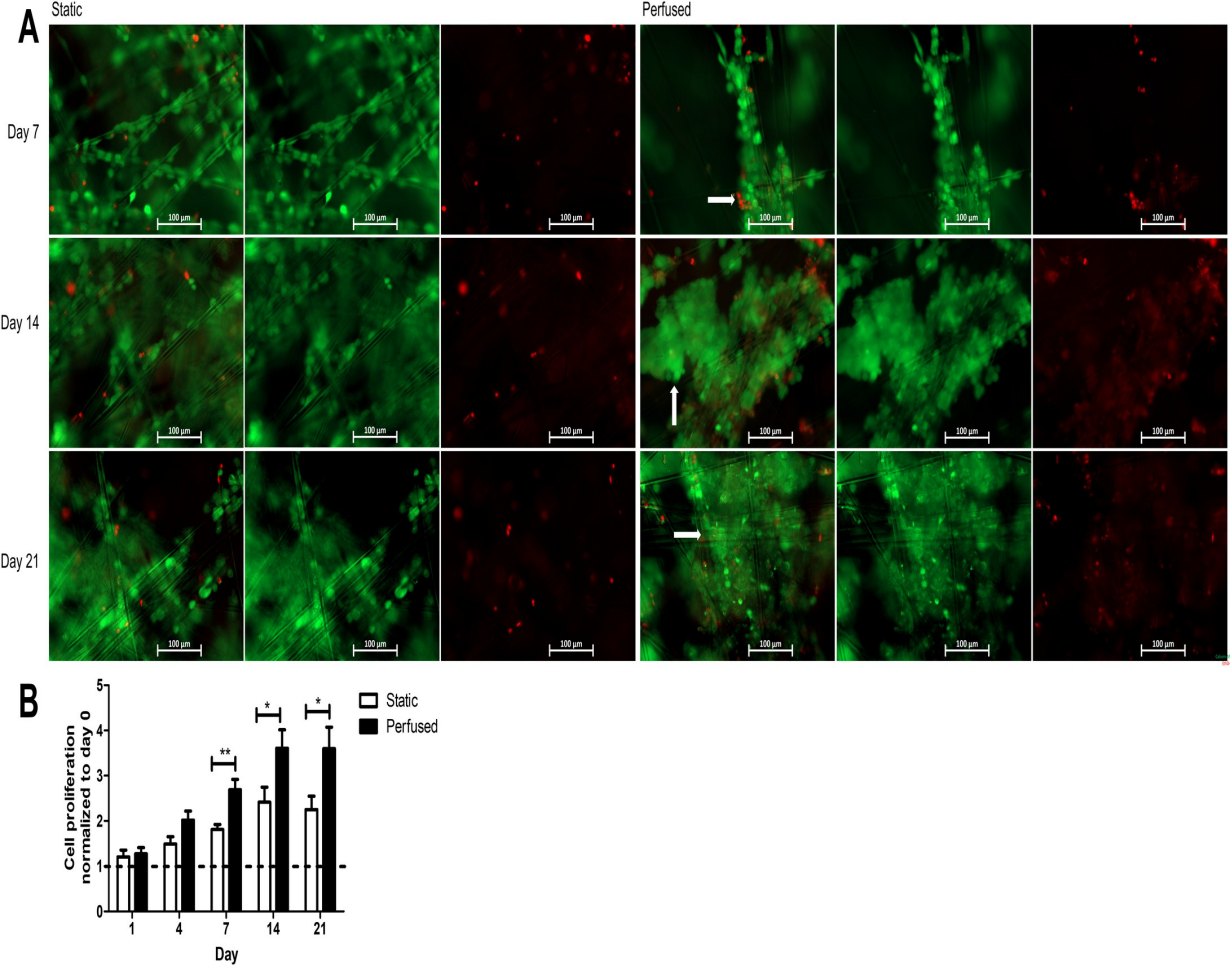
Effect of perfusion on cell proliferation in long term culture. MCF-7 cells were cultured in SeedEZ with or without perfusion for 21 days. (A) Representative images of cells stained with calcein-AM (green) and EthD-1 (red) at day 7, 14, 21. Scale bar = 100 μm. (B) Fluorescent intensity of GFP-labelled MCF-7 cells under static or perfused conditions normalized to signal at day 0. Statistical significance between static and perfused conditions. *p < 0.05, **p < 0.01.

Next, we hypothesised that perfusion would better support long term cell growth in 3D compared to static culture. To test this, we used MCF-7 cells stably transfected with GFP and maintained either in static or perfused conditions for up to 21 days. Cells tended to form aggregates under perfused culture conditions compared to that under static conditions. By day 7, cell growth was significantly increased under perfused conditions, evidenced by clusters of cells growing along the glass fibres of SeedEZ as indicated by the arrows in Fig 3A. When fluorescent signalling was quantified between cells cultured in static and perfused conditions, cell growth was significantly increased under perfused conditions by day 7 and this persisted to day 21 (Fig 3B).

### Effects of TAM under static and perfused conditions

**Fig 4:**
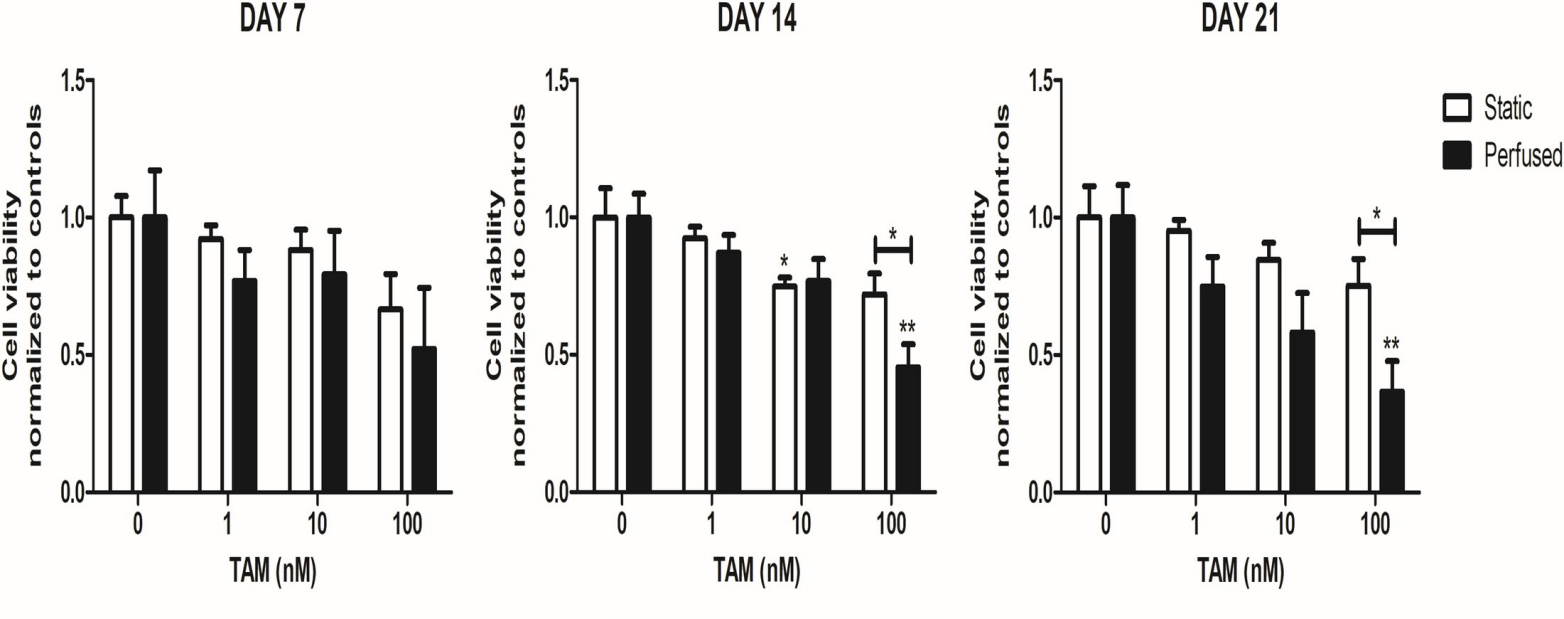
Response of GFP labelled MCF-7 cells to TAM. Cells were seeded on SeedEZ and cultured with or without PerfusionPal for 24 hours, then cells were treated with TAM under static or perfused conditions with cell viability measured by fluorescence intensity after 7, 14, and 21 days. Results represent the mean ± SD (n = 3). *p < 0.05, ** p < 0.01 between vehicle and treatment, or static versus perfused conditions (indicated by the line between the two conditions).

Having established that BC cell lines grew better under perfused condition in 3D both standard growth media and in epiFL, we next examined if perfusion would trigger an improved drug response to TAM (1–100 nM) [22, 23]. Due to supply issues with FibroLife (still ongoing), we were only able to do this in RPMI culture medium. MCF-7 cells were cultured for up to 21 days in static and perfused conditions in the presence of TAM. Response to TAM was similar in both static and perfused conditions until day 7, after which cells were more responsive under perfused conditions, especially with higher doses (Fig 4). With 100 nM TAM, there was a significantly enhanced inhibitory effect on cell viability under perfused condition compared to that of static culture after 14 days which persisted to 21 days.

### Perfusion culture supports growth and response to TAM in ER-positive patient-derived tumour-enriched explants in 3D

**Fig 5:**
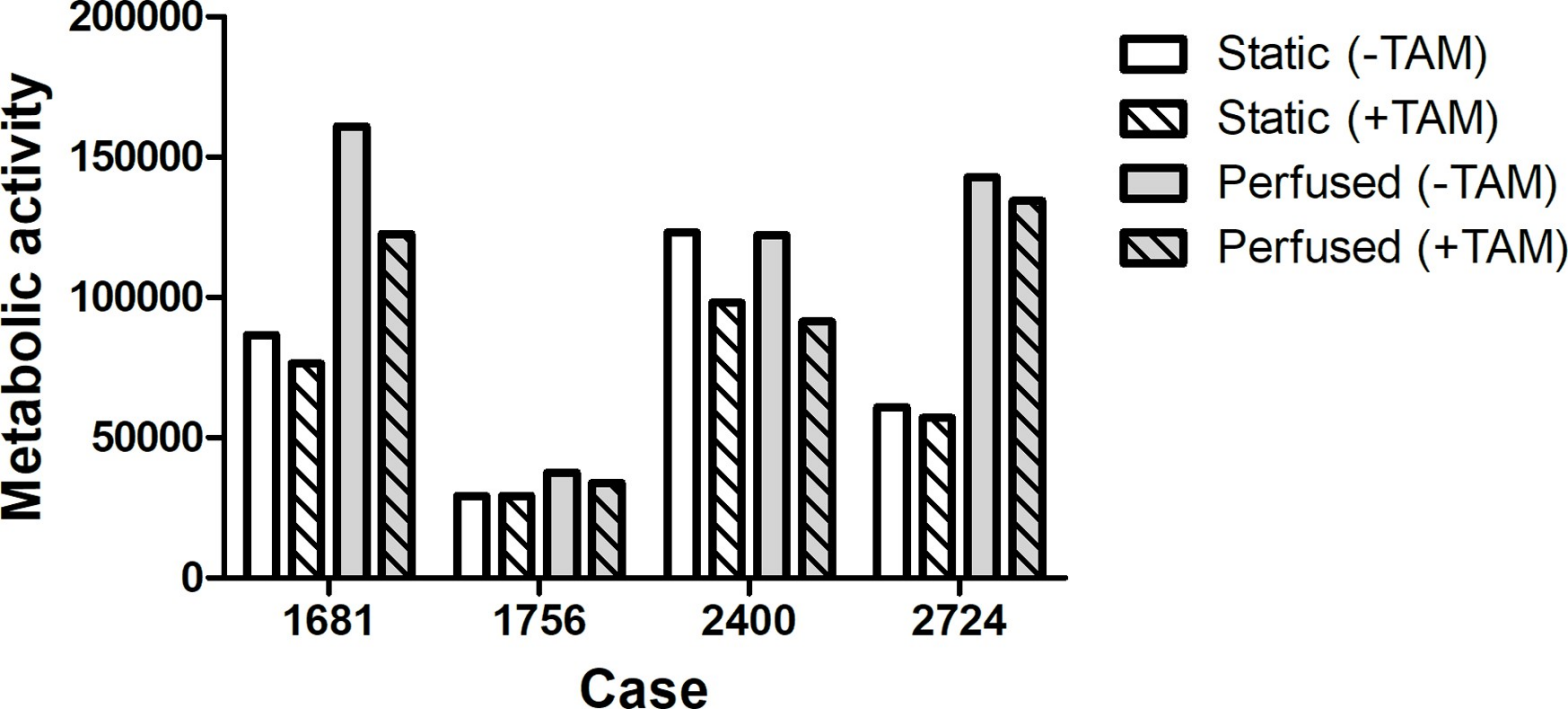
Response of patient-derived breast cancer explants to TAM under static or perfused conditions. Patient samples were seeded into SeedEZ scaffolds and cultured with or without perfusion. TAM (20 nM) was added after 24 hours, and samples were maintained for another 4 days. Metabolic activity was then analysed by AlamarBlue assay to compare static vs perfused conditions, using 0.1% v/v ethanol as the control in each condition.

Having shown that BC cell lines showed improved growth and response to TAM in 3D perfused cultures, we tested if this also applied to patient-derived tumour-enriched explants. Explant cultures were obtained from 4 primary ER-positive breast tumours from different patients and seeded into SeedEZ under static or perfused conditions. After 24 hours, TAM was introduced for 4 days. Metabolic activity was analysed by AlamarBlue assay. ER receptor status was also examined by immunofluorescence staining (S2 Fig). As shown in Fig 5 and improved metabolic activity of up to 2-fold was seen under perfused conditions in 3 out of 4 explants. In terms of drug response, when treated with TAM, sample 1681 showed a 12% reduction of metabolic activity under static culture; this doubled under perfused conditions. Cases 2400 and 2724, showed a similar inhibitory response in static and perfused conditions, while 1756 was insensitive towards TAM under static conditions but showed a modest reduction (10%) under perfused conditions.

## Discussion

The limitations of traditional 2D cell culture is now widely recognised and more sophisticated closer-to-patient cell culture systems are being developed and implemented [24]. Here, we advanced the use of PerfusionPal, a perfusable 3D culture system, to examine the effect of perfusion on viability and drug responses of BC cell lines, at the same time delivering a fully humanised method of breast cancer cell culture. This was extended further to explore the possibility of the system supporting the growth of *ex vivo* patient-derived BC samples.

Perfusion improved the homeostasis of the culture environment and increased cell metabolic activity/proliferation in a panel of BC cells, especially over long-term culture. This is in line with other studies. For example Pasini et al., demonstrated that MDA-MB-231 cells growing in 3D collagen scaffolds showed significant improvement in cell viability under perfused conditions after 7 days [25]. The perfusion system reported, generated continuous flow in a closed system, but only with bidirectional lateral cross-well perfusion. PerfusionPal, on the other hand, generates bidirectional in-well perfusion which is advantageous compared to one-way perfusion as all secretome/growth factors remain in the system. Additionally, PerfusionPal offered enhanced oxygen access from both apical and the basal sides of the cultures [20]. Other perfused systems have included a supporting matrix. SY5Y neuroblastoma cells cultured as microtumours in Matrigel showed increased viability and volume after 7 days in perfused culture under monodirectional flow compared to static, with long term culture of up to 35 days possible [26]. Similarly, NIH3T3 fibroblasts or EMT6 BC cells growing alone or in co-culture on silk fibroin scaffolds showed higher cell viabilities than in static culture condition over 21 days, with perfusion generated by agitation [27]. We did not use any matrix in our experiments as cells appeared to grow well in the naked SeedEZ scaffold. This brings advantages and disadvantages. It is worth considering integrating the extracellular matrix (ECM) components as their composition and organization can influence cell behaviour [28, 29], however most products used in 3D culture such as collagen and Matrigel have low and/or undefined mechanical strength which also degrades significantly over long-term culture [30], compared to the more stable glass fibre scaffold system we employed. Moreover, many of these biomaterials used as 3D culture matrices are of animal origin, which can result in batch variability, as well as posing environmental and ethical concerns. Further exploration could be done by coating SeedEZ with animal-free hydrogel such as peptide based hydrogels [31] to provide a 3D culture environment with controlled biochemical and mechanical properties.

Since our lab is working towards eliminating animal-derived components from cell culture, we fully humanised the PerfusionPal platform by using a modified cell culture medium devoid of animal-derived components, including FBS, termed epiFL. Our previous work has shown that culture in this medium stimulates the spontaneous transition of BC epithelial cells from growing as an adherent monolayer to loosely adherent spheroids which can then be disaggregated into single cell suspensions by mechanical dissociation [14]. Like our results in standard cell culture media, all three BC lines grown in epiFL showed higher metabolic activity under perfusion compared to static culture. As cell culture media is usually supplemented with FBS, using epiFL offers an animal-free alternative which can be added to the growing list of chemically defined or ‘humanized’ supplements for cell culture [32], providing scientists with opportunities to develop closer-to-patient model systems.

The benefits of perfusion extended to supporting primary cell culture as well. We assessed the response of 4 different patient-derived BC tumour-enriched explants under static or perfused culture condition. An increase in metabolic activity was observed in patient samples cultured under perfused compared to static conditions, ranging from 2.3-fold (case 2724), 1.9-fold (case 1681), 1.3-fold (case 1756). It has been reported in breast tissue slice models that dynamic culture conditions caused by agitation were essential to maintain the replicative potential of the cells [33]. Compared to static conditions, perfused culture of colorectal cancer fragments in a ‘sandwich’ collagen culture up to 3 days while preserved the architecture of the TME [34]. The same system was also applied successfully in culturing fragments of BC tissue for up to 3 weeks, with next generation sequencing confirming that perfusion not only improved the survival of all cell types in the TME but also maintained the gene signatures of the original tissue [35].

Our patient samples were all from ER-positive BC which accounts for around two thirds of all BC. It was encouraging to note that PerfusionPal supported the growth of primary BC tissues, particularly under perfused conditions. It is well known that developing patient derived xenograft (PDX) models from ER-positive BC is challenging; for ER-positive models, the estimated engraftment rate is less than 15% compared to triple negative BC at 30–80% [36]. Furthermore, PDX models require large numbers of animals for generation and maintenance. Hence, PerfusionPal could provide an alternative platform to support ER-positive BC overcoming the issues in generating and maintaining PDX, as well as positively addressing the 3Rs. However, unlike PDX models which can be expanded through successive transplantation, human tissue is a finite resource, hence one of the drawbacks of using patient-derived models is the limited ability to conduct experimental repeats due to small amounts of tissue available from individual patients after pathologists have used what is necessary for diagnostic purposes.

Our results support the possibility of using PerfusionPal in pre-clinical drug testing. We compared the therapeutic response of BC cell lines as well as primary patient samples cultured in 3D static and perfused conditions. In short term culture (< 2 weeks) no significant differences in drug response were observed between static and perfused conditions. However, in long term culture (> 2 weeks) MCF-7 cells were sensitized towards TAM in perfused condition. For patient samples, sample-specific responses were seen under perfused versus static conditions, which may be explained by sample heterogeneity between the 4 different tumours we evaluated.

PerfusionPal platform distinguishes itself from microfluidic devices that are typically used to study the interplay between flow and tumour cell behaviours. Due to the microscales, high pressure and flow rate are often needed to enhance oxygen availability in microfluidic devices. This may lead to high shear stress that could cause cellular injury and dilution of metabolites/signalling molecules [37]. Micro-channels are also easy to trap gas or clog as proteins accumulate to the interior of the channels which hinders consistent operation especially for long term culture. Consequently, culturing patient tissue in a macro scale has begun to be appreciated more for personalized drug testing as this allows use of larger amounts of native patient tumour tissues, to perverse the intra-tumour genetic/cellular heterogeneity [38] present in almost all cancer lesions including BC [39]. The preservation/recapitulation of TME is more challenging using microfluidics without losing the high degree of heterogeneity of parental primary tissues due to restrictions in what these devices can hold, physically.

In conclusion, we validated a perfused 3D cell culture platform which not only supports the growth of BC cell lines cultured in fully humanised condition but also *ex vivo* patient-derived BC tumour-enriched samples. These hold promise for future testing of treatment responses, overcoming some of the ethical and scientific disadvantages of using animal models in BC research.

## Supporting information

S1 Table Biological features and culture conditions of breast cancer cell lines used in this study. (DOCX)

S1 Fig Scheme of the PerfusionPal system. (A) Photograph of the assembled PerfusionPal with pump. (B) Exploded diagram of the 12-well system showing the three components (lid, multi-well insert, and reservoir) (Left) as well as the side view (Top Right)/ top view (Bottom Right) of the assembled system. (C) An illustration of the PerfusionPal plate attached to a syringe pump to infuse and withdraw blood substitute (transparent, in figure coloured blue), which subsequently pushes culture media with a controlled flow through the SeedEZ scaffold hosting cells in 3D.
(DOCX)

S2 Fig Immunofluorescence staining of ERα in primary patient samples cultured in PerfusionPal. Patient samples were seeded onto SeedEZ scaffolds and cultured with perfusion for 5 days. SeedEZ with cells were then washed 3 times with phosphate-buffered saline (PBS) gently prior to fixing in 4% paraformaldehyde (PFA). These scaffolds were then blocked with 2% bovine serum albumin (BSA) in PBS and incubated with ERα antibody (1D5, Invitrogen, Waltham, USA) with 1:400 dilution at 4°C overnight. After which, scaffolds were incubated with secondary AlexaFluor 488 (A21202, Invitrogen, Waltham, USA) incubated for 1 hour at room temperature, then mounted to glass slides with mounting medium containing DAPI (Abcam, Cambridge, UK). Scaffolds were imaged using a Zeiss Axio Observer Z1 inverted microscope. Monolayer cultures of MCF-7 cells were used as positive controls; negative controls were generated by staining whilst omitting the primary antibody. Immunofluorescence showed that nuclear ER positivity of the patient derived explants were maintained in perfused condition. However sharp focus was difficult to achieve in 3D and the glass fibre scaffolds partly obscured the staining in some cases (e.g. 1756).
(DOCX)
