# Supplementary material for: Validation of a 3D perfused cell culture platform as a tool for humanised preclinical drug testing in breast cancer using established cell lines and patient-derived tissues
Source: PLoS One. 2023 Mar 16;18(3):e0283044. doi: 10.1371/journal.pone.0283044 (PMC10019722; doi:10.1371/journal.pone.0283044)
Supplement: S1 Table — (DOCX) [file pone.0283044.s001.docx]

S1 Table. Biological features and culture conditions of breast cancer cell lines used in this study.

| Cell line | Classification(40) | ER | PR | HER2 | Culture medium |
| --- | --- | --- | --- | --- | --- |
| MCF-7 | Luminal A | + | + | - | RPMI + 5% FCS |
| BT-474 | Luminal B | + | + | + | RPMI + 10 % FCS |
| MDA-MB-231 | Triple negative B | - | - | - | DMEM + 10 % FCS |
