# Supplementary material for: Validation of a 3D perfused cell culture platform as a tool for humanised preclinical drug testing in breast cancer using established cell lines and patient-derived tissues
Source: PLoS One. 2023 Mar 16;18(3):e0283044. doi: 10.1371/journal.pone.0283044 (PMC10019722; doi:10.1371/journal.pone.0283044)
Supplement: S1 Fig — (A) Photograph of the assembled PerfusionPal with pump. (B) Exploded diagram of the 12-well system showing the three components (lid, multi-well insert, and reservoir) (Left) as well as the side view (Top Right)/ top view (Bottom Right) of the assembled system. (C) An illustration of the PerfusionPal plate attached to a syringe pump to infuse and withdraw blood substitute (transparent, in figure coloured blue), which subsequently pushes culture media with a controlled flow through the SeedEZ scaffold hosting cells in 3D. (DOCX) [file pone.0283044.s002.docx]

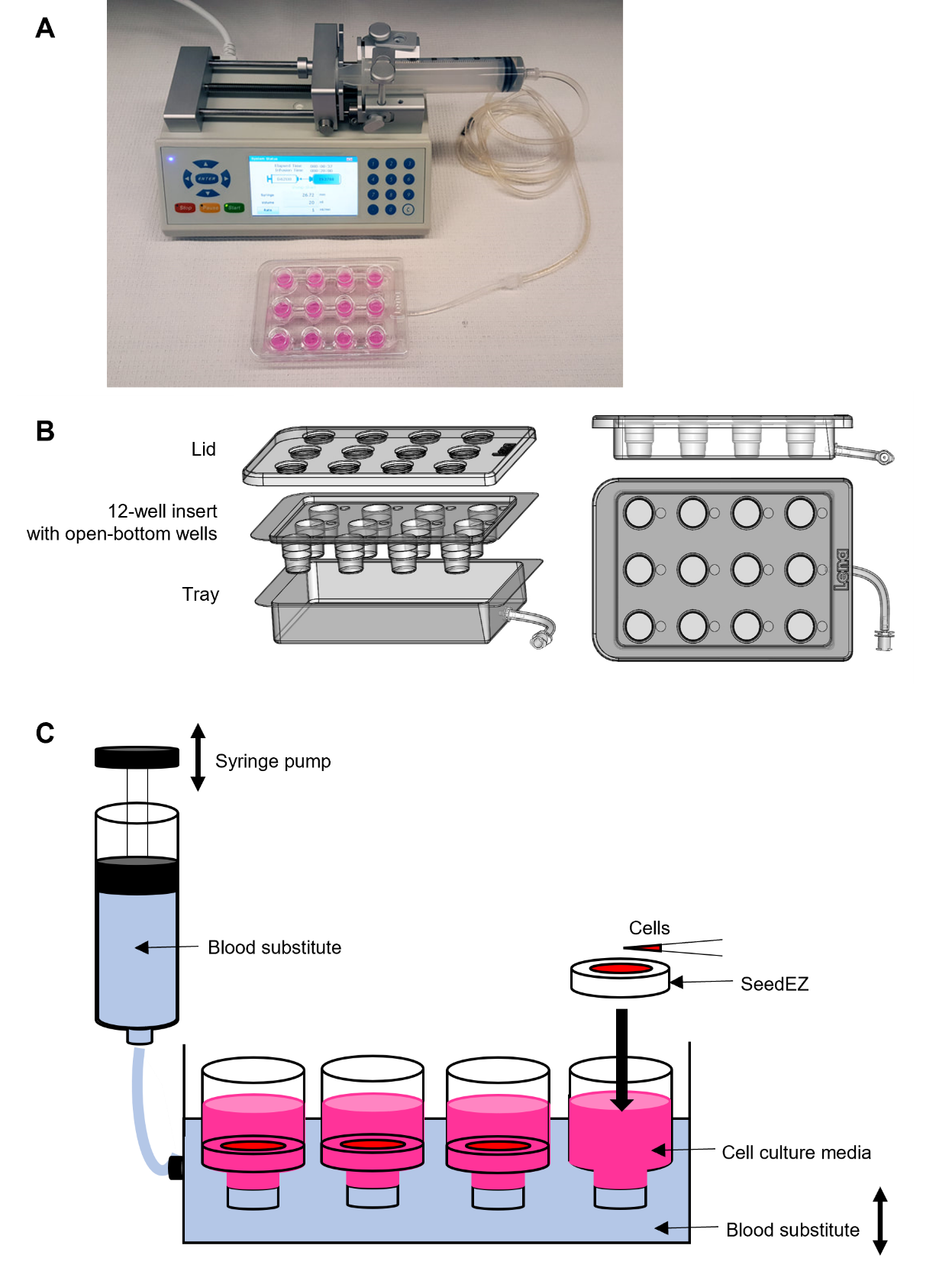


S1 Fig. Scheme of the PerfusionPal system. (A) Photograph of the assembled PerfusionPal with pump. (B) Exploded diagram of the 12-well system showing the three components (lid, multi-well insert, and reservoir) (Left) as well as the side view (Top Right)/ top view (Bottom Right) of the assembled system. (C) An illustration of the PerfusionPal plate attached to a syringe pump to infuse and withdraw blood substitute (transparent, in figure coloured blue), which subsequently pushes culture media with a controlled flow through the SeedEZ scaffold hosting cells in 3D.
