# Supplementary material for: Validation of a 3D perfused cell culture platform as a tool for humanised preclinical drug testing in breast cancer using established cell lines and patient-derived tissues
Source: PLoS One. 2023 Mar 16;18(3):e0283044. doi: 10.1371/journal.pone.0283044 (PMC10019722; doi:10.1371/journal.pone.0283044)
Supplement: S2 Fig — Patient samples were seeded onto SeedEZ scaffolds and cultured with perfusion for 5 days. SeedEZ with cells were then washed 3 times with phosphate-buffered saline (PBS) gently prior to fixing in 4% paraformaldehyde (PFA). These scaffolds were then blocked with 2% bovine serum albumin (BSA) in PBS and incubated with ERα antibody (1D5, Invitrogen, Waltham, USA) with 1:400 dilution at 4°C overnight. After which, scaffolds were incubated with secondary AlexaFluor 488 (A21202, Invitrogen, Waltham, USA) incubated for 1 hour at room temperature, then mounted to glass slides with mounting medium containing DAPI (Abcam, Cambridge, UK). Scaffolds were imaged using a Zeiss Axio Observer Z1 inverted microscope. Monolayer cultures of MCF-7 cells were used as positive controls; negative controls were generated by staining whilst omitting the primary antibody. Immunofluorescence showed that nuclear ER positivity of the patient derived explants were maintained in perfused condition. However sharp focus was difficult to achieve in 3D and the glass fibre scaffolds partly obscured the staining in some cases (e.g. 1756). (DOCX) [file pone.0283044.s003.docx]

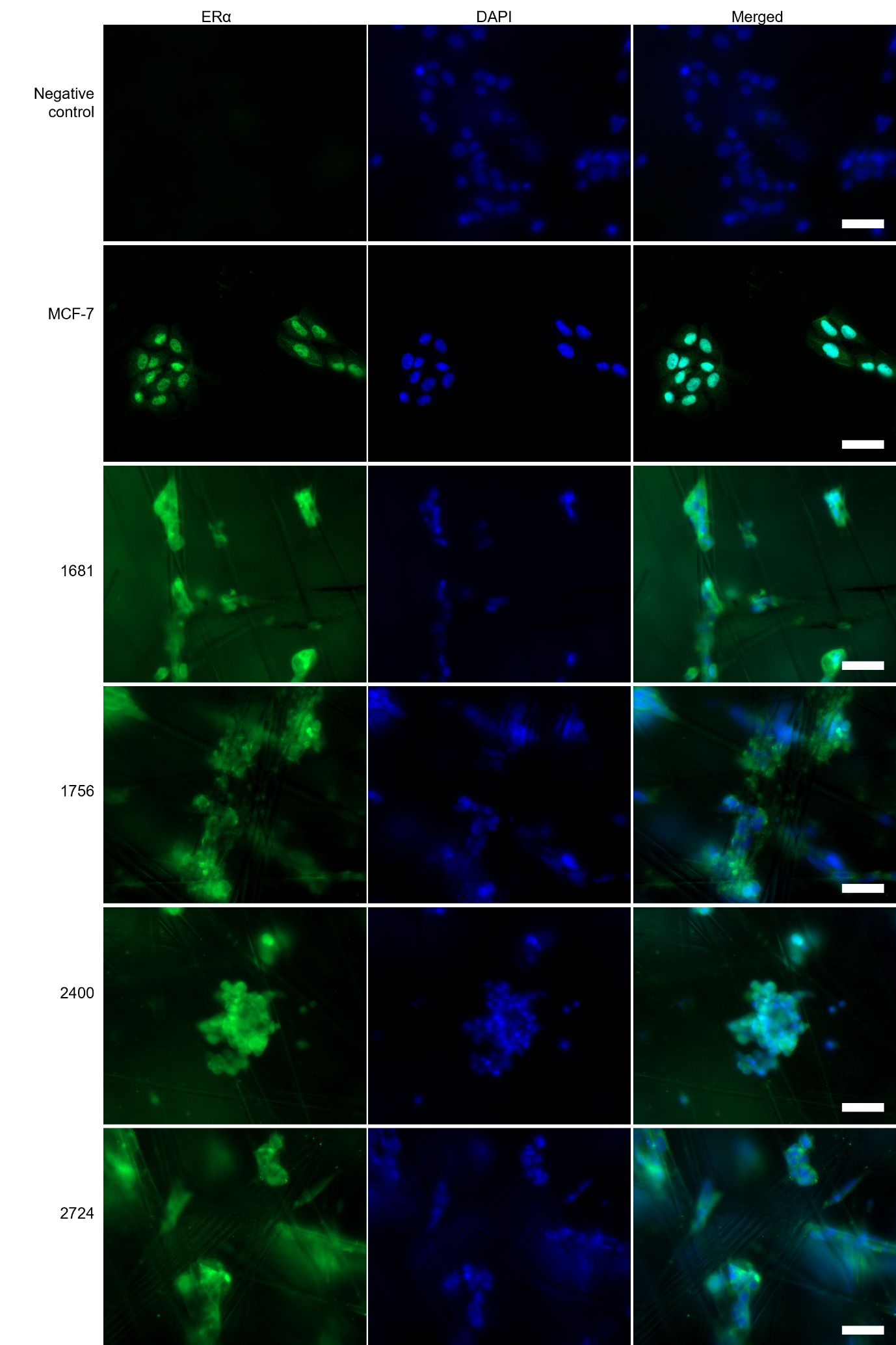


S2 Fig. Immunofluorescence staining of ERα in primary patient samples cultured in PerfusionPal. Patient samples were seeded onto SeedEZ scaffolds and cultured with perfusion for 5 days. SeedEZ with cells were then washed 3 times with phosphate-buffered saline (PBS) gently prior to fixing in 4% paraformaldehyde (PFA). These scaffolds were then blocked with 2% bovine serum albumin (BSA) in PBS and incubated with ERα antibody (1D5, Invitrogen, Waltham, USA) with 1:400 dilution at 4°C overnight. After which, scaffolds were incubated with secondary AlexaFluor 488 (A21202, Invitrogen, Waltham, USA) incubated for 1 hour at room temperature, then mounted to glass slides with mounting medium containing DAPI (Abcam, Cambridge, UK). Scaffolds were imaged using a Zeiss Axio Observer Z1 inverted microscope. Monolayer cultures of MCF-7 cells were used as positive controls; negative controls were generated by staining whilst omitting the primary antibody. Immunofluorescence showed that nuclear ER positivity of the patient derived explants were maintained in perfused condition. However sharp focus was difficult to achieve in 3D and the glass fibre scaffolds partly obscured the staining in some cases (e.g. 1756).
